# Identification, Molecular Characteristics, and Evolution of YABBY Gene Family in *Melastoma dodecandrum*

**DOI:** 10.3390/ijms24044174

**Published:** 2023-02-20

**Authors:** Jie Huang, Gui-Zhen Chen, Sagheer Ahmad, Qin Wang, Song Tu, Xiao-Ling Shi, Yang Hao, Yu-Zhen Zhou, Si-Ren Lan, Zhong-Jian Liu, Dong-Hui Peng

**Affiliations:** Key Laboratory of National Forestry and Grassland Administration for Orchid Conservation and Utilization at College of Landscape Architecture and Art, Fujian Agriculture and Forestry University, Fuzhou 350002, China

**Keywords:** the YABBY gene family, subcellular localization, growth and development, phylogeny analysis, genome-wide identification

## Abstract

The YABBY gene family plays an important role in plant growth and development, such as response to abiotic stress and lateral organ development. *YABBY* TFs are well studied in numerous plant species, but no study has performed a genome-wide investigation of the YABBY gene family in *Melastoma dodecandrum*. Therefore, a genome-wide comparative analysis of the YABBY gene family was performed to study their sequence structures, cis-acting elements, phylogenetics, expression, chromosome locations, collinearity analysis, protein interaction, and subcellular localization analysis. A total of nine *YABBY* genes were found, and they were further divided into four subgroups based on the phylogenetic tree. The genes in the same clade of phylogenetic tree had the same structure. The cis-element analysis showed that *MdYABBY* genes were involved in various biological processes, such as cell cycle regulation, meristem expression, responses to low temperature, and hormone signaling. *MdYABBYs* were unevenly distributed on chromosomes. The transcriptomic data and real-time reverse transcription quantitative PCR (RT-qPCR) expression pattern analyses showed that *MdYABBY* genes were involved in organ development and differentiation of *M. dodecandrum*, and some *MdYABBYs* in the subfamily may have function differentiation. The RT-qPCR analysis showed high expression of flower bud and medium flower. Moreover, all *MdYABBYs* were localized in the nucleus. Therefore, this study provides a theoretical basis for the functional analysis of YABBY genes in *M. dodecandrum*.

## 1. Introduction

Transcription factors (TFs) are critical in regulating plant growth, development, and response to stress [1]. YABBY is a family of TFs belonging to the zinc finger protein superfamily [2]. YABBY genes play crucial roles in the formation of lateral organs during plant growth and development [3,4,5,6]. The YABBY family contains two typical domains, the YABBY domain in the C-terminus and the C2C2 zinc finger domain in the N-terminus [2,7]. *Arabidopsis thaliana* contains six YABBY genes, namely *YABBY1*, *YABBY2*, *YABBY3*, *YABBY4*, *YABBY5*, and *CRC* [8]. They can be divided into five subfamilies: FIL, CRC, INO, YABBY2, and YABBY5 [9,10]. Eight YABBY genes are found in *Oryza sativa*, but there are only four subfamilies in rice, and there is no YABBY5 subfamily [11].

The functional studies of the YABBY gene family show that it plays indispensable roles in the establishment of the dorsal-ventral polarity of plant tissue, leaves and leaf-derived organs development, plant morphogenesis, flower formation, fruit development, and plant biotic and abiotic stress processes [12,13,14]. In *A. thaliana*, CRC is involved in the development of nectary and distal carpel. INO promotes the development of the outer integument of ovule to the seed coat [15]. The CRC and INO subfamily genes are flower-specific genes, which have specific expression in carpels and ovules; FIL/YAB3, YAB2, and YAB5 subfamily genes are vegetative growth genes, which have specific expression in leaves, cotyledons, and floral organs [12,14,16,17,18,19]. In *O. sativa*, *OsYAB1* regulates the differentiation of some specific cell types [7,11]. In addition, *OsYAB1* participates in the feedback regulation of GA biosynthesis [20]. A *GmFILa* transcription factor from soybean has been identified, which belongs to the FIL subfamily [14]. The mRNA blotting analysis shows that *GmFILa* is explicitly expressed in leaves and flower bud primordia. The overexpression of this gene in *A. thaliana* results in the change of the dorsal and ventral polarity of epidermis leaf tissues, the prolongation of fluorescence, and the inhibition of apical meristem development in transgenic plants [14]. In addition, studies have shown that YABBY genes are involved in the biosynthesis of plant secondary metabolism [12,21]. For example, *Arabidopsis* FIL regulates anthocyanin biosynthesis [21]. *AaYABBY5* in *Artemisia annua* (*Artemisia*, Asteraceae) controls artemisinin biosynthesis by enhancing the activity of *CYP71AV1* [12].

*M. dodecandrum* is a species of *Melastoma* in Melastomataceae. Plants of *Melastoma* possess great ornamental value, and their fruits can be used for food and medicinal purposes. Recently, the YABBY gene family has been discussed for *A. thaliana*, *Vitis vinifera*, *Glycine max*, *Punica granatum*, *Mangifera indica*, *Lactuca sativa*, *Triticum aestivum*, *Juglans regia*, and *J. mandshurica* [2,8,22,23,24,25]. The availability of *M. dodecandrum* genome has made it convenient to identify gene families [26]. Therefore, we report the YABBY gene family of *M. dodecandrum*. This study gives new insights into the role of the YABBY genes in the growth and development of *M. dodecandrum*.

## 2. Result

### 2.1. Identification and Characterization of the YABBY Gene Family in M. dodecandrum

**Figure 1:**
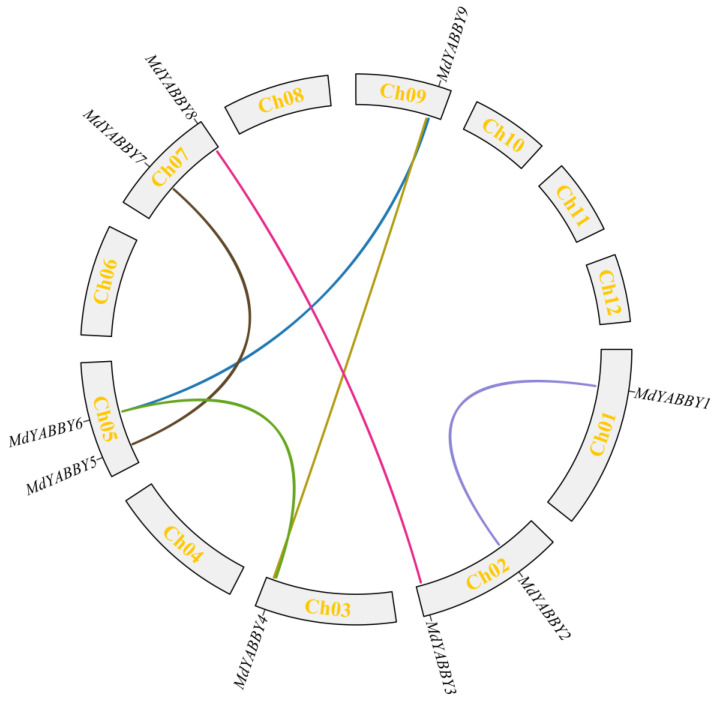
Gene locations and intra-specific synteny relationships of the YABBY gene family in *M. dodecandrum*. Lines denote syntenic YABBY gene pairs of *M. dodecandrum* on the chromosomes.

A total of 9 YABBY genes were unevenly distributed on 6 chromosomes (total 12 chromosomes) of *M. dodecandrum* genome. According to their chromosome location information, nine YABBY genes were named *MdYABBY1*−*MdYABBY9* (Figure 1). The characterization of the YABBY gene family was analyzed by the ExPASy website. The result showed that the isoelectric point (PI) of YABBY proteins in *M. dodecandrum* ranged from 8.42 (*MdYABBY4*) to 9.66 (*MdYABBY2*) (Table 1). All of the *MdYABBYs* exhibited a PI higher than 8.00. The molecular weight was ranged from 14360.52 Da (*MdYABBY5*) to 29200.37 Da (*MdYABBY2*). The number of amino acids was ranged from 128 aa (*MdYABBY5*) to 263 aa (*MdYABBY2*), and the aliphatic index was from 60.20 to 79.77. Further study found that the Grand average of hydropathicity (GRAVY) of MdYABBY proteins was negative (ranging from −0.563 to −0.270). Therefore, MdYABBY proteins are hydrophilic proteins, but with different degrees of hydrophilicity. Among the nine MdYABBY proteins, only *MdYABBY7* and *MdYABBY5* showed an instability coefficient of less than 40, which suggested that most MdYABBY proteins were unstable. The subcellular localization prediction showed that all the *MdYABBY* genes were located in the nucleus.

### 2.2. Phylogeny Analysis of the YABBY Gene Family

**Figure 2:**
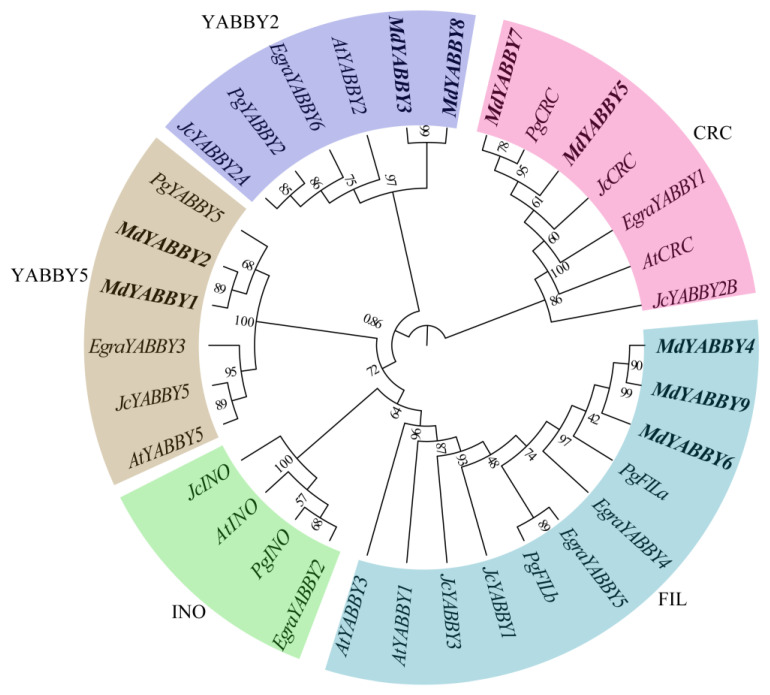
Phylogeny analysis in *E. grandis*, *M. dodecandrum*, *J. curcas*, *P. granatum*, and *A. thaliana*. The genes begin with “At” to represent the genes of *A. thaliana*, “Md” to represent the genes of *M. dodecandrum*, “Pg” to represent the genes of *P. granatum*, “Eg” to represent the genes of *E. grandis*, and “Jc” to represent the genes of *J. curcas*.

To explore the phylogenetic relationship and the evolutionary pattern of the YABBY genes in *M. dodecandrum*, a total of 34 YABBY genes from *M. dodecandrum* (9), *A. thaliana* (6), *P. granatum* (6), *J. curcas* (7), and *E. grandis* (6) were used to construct the phylogenetic tree. The result showed that the 34 YABBY genes could be divided into five clades (FIL, INO, CRC, YABBY2, and YABBY5), while the YABBY genes in *M. dodecandrum* lacked the INO clade (Figure 2). Among the five clades, the FIL clade was the largest clade, containing 11 YABBY genes, including three MdYABBYs, and two YABBY genes from other species. The CRC clade and the YABBY2 clade all contained one AtYABBY, one PgYABB, two JcYABBYs, one EgYABBY, and two MdYABBYs. The YABBY5 clade had one AtYABBY, one PgYABBY, one JcYABBY, one EgYABBY, and two MdYABBYs. The clade INO contained four YABBYs. Clade YABBY2 had six YABBY genes.

### 2.3. Gene Structure Analysis of the YABBY Gene Family

**Figure 3:**
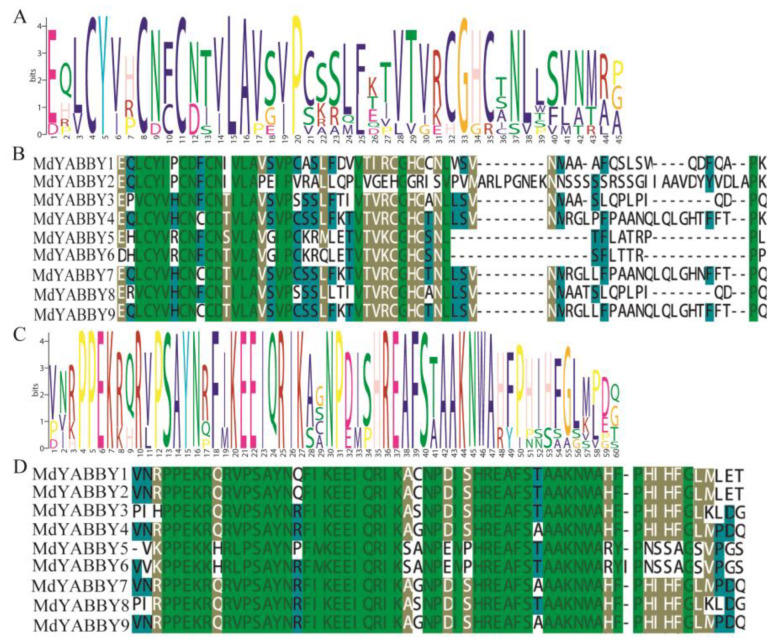
Conserved domains and Seqlogos of the YABBY gene family in *M. dodecandrum*. (**A**) Sequence logo of the conserved C2C2 domain (motif 2); (**B**) conserved sequence alignment of the C2C2 domain; (**C**) sequence logo of the conserved YABBY domain (motif 1); (**D**) conserved sequence alignment of the YABBY domain.

**Figure 4:**
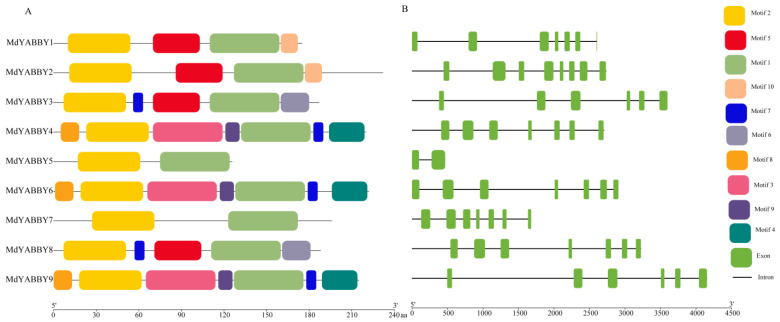
Conserved motifs and exon−intron structures of the YABBY gene family in *M. dodecandrum.* (**A**) Conserved motifs of MdYABBY; (**B**) exon−intron structures of MdYABBY genes.

The conserved domain analysis showed that all YABBY genes of *M. dodecandrum* had two typical domains: the C2C2 domain (Figure 3A,B) and the YABBY domain (Figure 3C,D). The conservative motifs of MdYABBYs were predicted by the MEME website, and the result was visualized by TBtools. A total of 10 motifs were identified (Figure 4A). The result showed that the nine MdYABBY proteins had conserved motifs 1 (YABBY domain) and 2 (C2C2 domain) (Figure 4A). MdYABBY4, MdYABBY6, and MdYABBY9 had the most motifs—a total of seven motifs, followed by MdYABBY1 and MdYABBY2 with four motifs, while MdYABBY5 and MdYABBY7 contained only two motifs. The MdYABBYs in the same clade in the phylogenetic tree (Figure 2) had the same motifs and arrangement order. Motif 10 was only present in clade YABBY5. Motifs 3, 4, 5, 7, 8, and 9 were only found in clade FIL. Motif 6 only existed in clade YABBY2. Motif 5 was found in clades YABBY2 and YABBY5.

The exon−intron structure is one of the important evolutionary features of genes, which provides an important basis for the study of its functional diversification. In this study, the gene structure of the YABBY gene family was performed by using the GFF annotation file of *M. dodecandrum* genome in TBtools. The result showed that most *MdYABBY* genes had seven exons (Figure 4B). *MdYABBY2* had eight exons. *MdYABBY3* and *MdYABBY9* had six exons. *MdYABBY5* had only two exons and one intron. *MdYABBY2* had seven introns, *MdYABBY3* and *MdYABBY9* had five introns, and the rest *MdYABBYs* had six introns.

### 2.4. Cis-Acting Element Prediction of the YABBY Gene Family in M. dodecandrum

**Figure 5:**
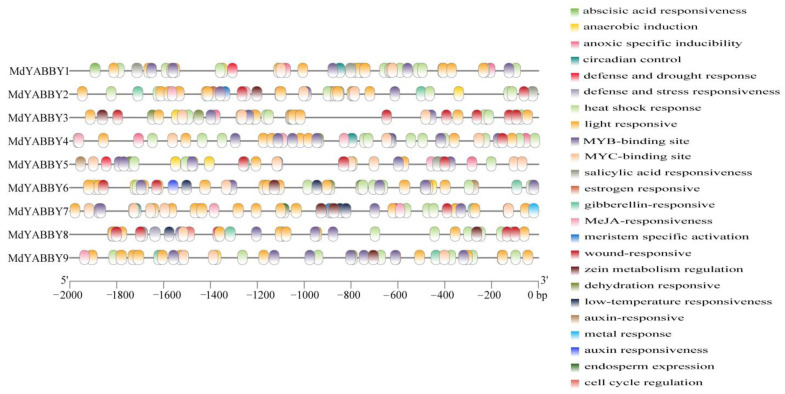
Types and numbers of cis-acting elements in promoters of *M. dodecandrum* in the YABBY gene family.

A total of 665 cis-acting elements of the YABBY gene family were predicted in *M. dodecandrum*. In addition to a large number of essential elements, i.e., CAAT-box and TATA-box, other regulatory elements of the *MdYABBY* gene family were also highly abundant, such as abscisic acid responsiveness, auxin-responsive elements, MYB-binding sites, light-responsive elements, and MeJA responsiveness. The prediction results showed that all genes had 2–11 abscisic-acid-responsiveness elements, 4–18 light responsive elements, 5–10 MYB-binding sites, 2–8 MeJA-responsiveness elements, and 1–8 MYC-binding sites. Moreover, 88.9% of *MdYABBY* genes had 1–3 dehydration-responsiveness elements, 1–3 gibberellin-responsiveness elements, and 1–3 low-temperature-responsiveness elements. A proportion of 77.8% of *MdYABBY* genes had 1–6 enhancer-like elements involved in anoxic specific inducibility and anaerobic induction. A proportion of 66.7% of *MdYABBY* genes had 1–2 auxin-responsive elements and 1–3 zein metabolism-regulation elements. A proportion of 55.6% of *MdYABBY* genes had 1–2 salicylic-acid-responsiveness elements. A proportion of 44.4% of genes had 1–3 MYBHv1-binding sites, 1–3 meristem-expression elements, and one circadian control elements. A proportion of 33.3% of genes had one cell-cycle-regulation element and one defense and stress-responsiveness element. Only *MdYABBY7* contained endosperm-expression elements (Figure 5).

### 2.5. Gene Location and Collinearity Analysis

The gene location analysis showed that the nine *MdYABBY* genes were unevenly distributed on the chromosomes of the genome (Figure 1). *MdYABBY1* was located on chromosome 1, *MdYABBY2* and *MdYABBY3* were distributed on chromosome 2, *MdYABBY4* was located on chromosome 3, *MdYABBY5* and *MdYABBY6* were distributed on chromosome 5, *MdYABBY7* and *MdYABBY8* were distributed on chromosome 7, and *MdYABBY9* was located on chromosome 9, while chromosomes 4, 6, 8, 10, 11, and 12 did not have *MdYABBY* genes. 

The intra-species synteny analysis was performed by TBtools to investigate the tandem replication and fragment replication events in *M. dodecandrum* (Figure 1). The result showed that there was no tandem replication in the MdYABBY gene family. Six pairs of fragment replication genes were found in *MdYABBYs*, including *MdYABBY1*/*MdYABBY2*, *MdYABBY3*/*MdYABBY8*, *MdYABBY4*/*MdYABBY6*, *MdYABBY4*/*MdYABBY9*, *MdYABBY5*/*MdYABBY7*, and *MdYABBY6*/*MdYABBY9* (Figure 1). Furthermore, gene duplication occurred more frequently in chromosome 5 than in the other chromosomes of *M. dodecandrum*. To estimate the evolutionary constraints between duplicated gene pairs, the Ka and Ks parameters were calculated. The Ka of *MdYABBYs* ranged from 0.02688089 to 0.15915999, the Ks ranged from 0.13608643 to 0.91519305, and the Ka/Ks ratio ranged from 0.07895371 to 0.19752809, suggesting that they underwent strong purifying selection during evolution (Table 2).

**Figure 6:**
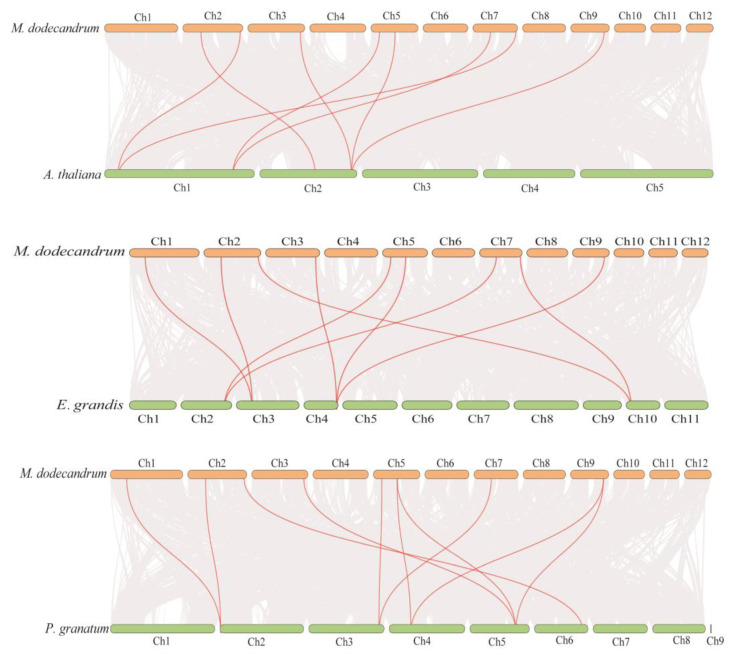
Synteny analysis of the YABBY gene family between *M. dodecandrum* and *A. thaliana*, *E. grandis*, and *P. granatum*. The red lines represent the colinear YABBY gene pairs.

What’s more, to analyze the potential evolutionary processes of the MdYABBY gene family, we investigated the collinear relationship between *M. dodecandrum* and three species, including *A. thaliana*, *E. grandis*, and *P. granatum* (Figure 6). The result showed that *M. dodecandrum* exhibited more orthologous pairs with *P. granatum* (ten pairs) than with *A. thaliana* (eight pairs) and *E. grandis* (nine pairs). Among these gene pairs, seven *M. dodecandrum* YABBY genes exhibited collinear relationships with *A. thaliana*, *E. grandis*, and *P. granatum.*

### 2.6. Expression Patterns of YABBY in M. dodecandrum 

**Figure 7:**
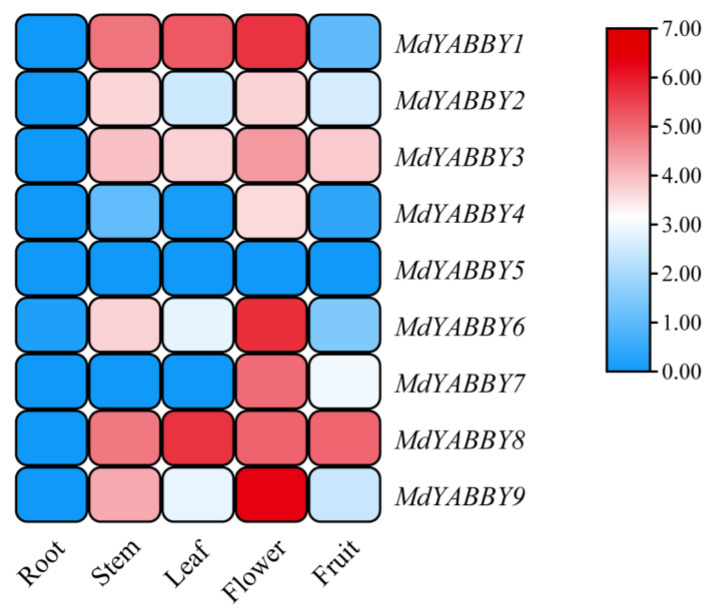
Heatmap of the YABBY gene family in *M. dodecandrum*. The color scale at the right of the heatmap refers to the relative expression level, and the color gradient from faint blue to red represents the increasing expression level.

All the *MdYABBY* genes showed low expression in the root, and *MdYABBY5* showed low expression in all organs. *MdYABBY9* and *MdYABBY6* showed high and medium expression in the flower and stem, respectively, but low expression in the leaf, root, and fruit. *MdYABBY4* was highly expressed in the flower but showed low expression in the other organs. *MdYABBY7* was significantly expressed in flowers, generally expressed in the fruit, and showed low expression in the other organs. *MdYABBY2* was highly expressed in the flower and stem, normally expressed in leaf and fruit, and showed low expression in the root. Elevated expression of *MdYABBY1* was observed in the flower, leaf, and stem, but a low expression was observed in the root and fruit. *MdYABBY3* and *MdYABBY8* showed significant expression in the flower, leaf, fruit, and stem, but low expression was noticed in the root (Figure 7).

**Figure 8:**
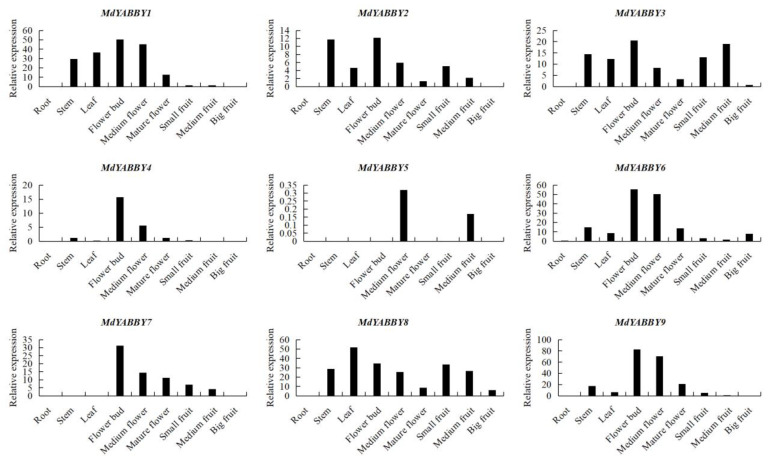
The expression patterns of the *MdYABBY* genes family in *M. dodecandrum* based on RT-qPCR.

The tissue-specific expression was further evaluated (Figure 8). The RT-qPCR result showed that most *MdYABBY* genes were expressed in multiple tissues and some showed similar expression trends in different tissues. *MdYABBY1*−*MdYABBY9* had a low expression level in the root and big fruit. *MdYABBY6* and *MdYABBY9* were expressed in stem, leaf, flower, and fruit, and the highest expression was observed in the flower bud and medium flower. The expression level of *MdYABBY4* was high in the flower bud, and a medium expression was seen in medium flower. It had low expression in leaf, stem, mature flower, and different stages of fruit development. *MdYABBY1* had the maximum expression in the flower bud, followed by medium flower, and medium expression was observed in leaf, stem, and mature flower. The expression level of *MdYABBY7* gradually decreased during fruit and flower development, while it was not expressed in stem, root, and leaf. *MdYABBY2* and *MdYABBY8* also showed a decreasing expression trend during fruit and flower development. *MdYABBY3* showed a trend of an initial increase followed by a decrease during fruit and flower development. *MdYABBY5* was only expressed in medium flower and medium fruit. 

### 2.7. Protein Subcellular Localization Analysis of the YABBY Gene Family

**Figure 9:**
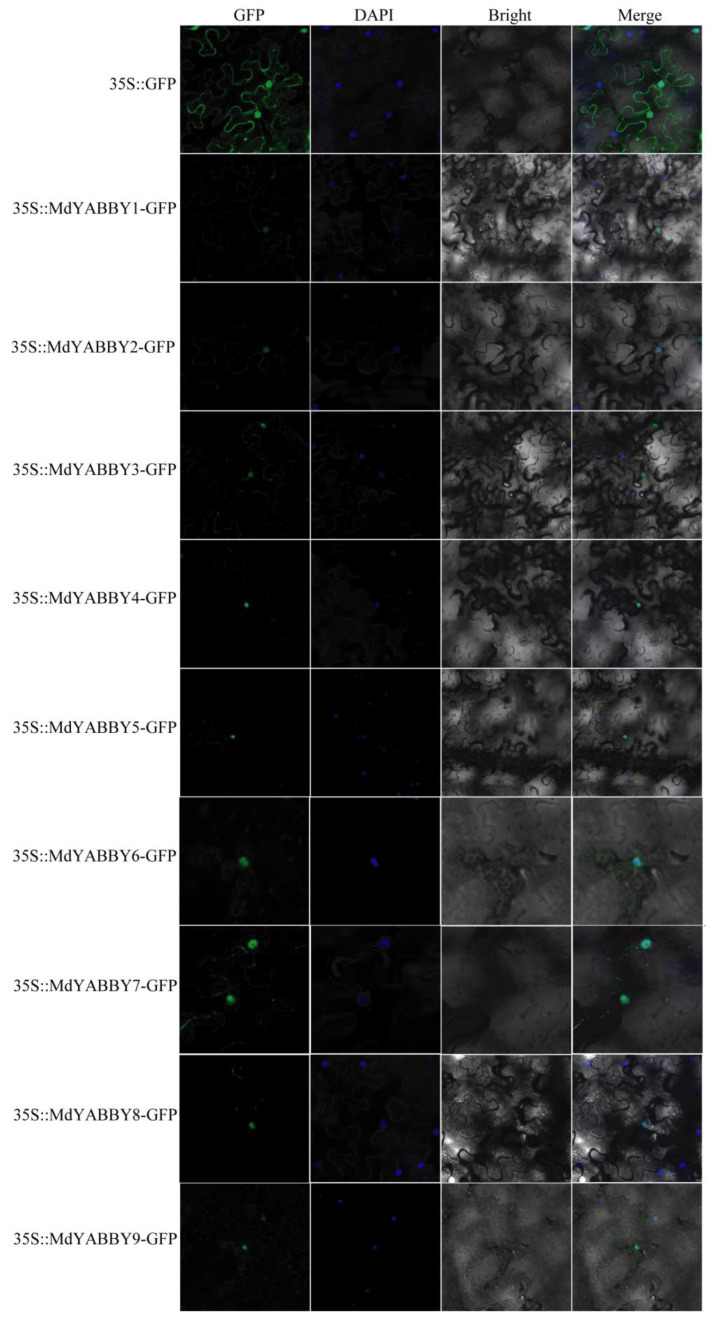
Subcellular localization of *MdYABBYs*. Scale bars = 10 µm. 35S::GFP was used as a control.

In this study, all MdYABBY proteins were predicted to target the nucleus by CELLO v2.5 (Table 1). To identify the subcellular localization of MdYABBY proteins, we cloned the *MdYABBYs* and introduced them into the pMDC202 vector by a CaMV-35S promoter. Then, we transiently co-expressed the MdYABBYs−GFP fusion protein in *Nicotiana benthamiana* leaves. The green fluorescence of the protein expressed by the pMDC202−GFP control vector was visible on the cell membrane and nucleus (Figure 9). However, the green fluorescence of the protein expressed by pMDC202−MdYABBYs−GFP fusion vector was only distributed in the nucleus, consisting of the nuclear marker (DAPI). The results of subcellular localization (Figure 9) are consistent with the results predicted by the website (Table 1). The result showed that the *MdYABBYs* were nuclear proteins (Table 1).

## 3. Discussion

The YABBY gene family belonging to the zinc finger protein superfamily mainly affects the development of leaf and flower organs, stress responses, and lateral organs development [27]. In present study, the YABBY genes of *M. dodecandrum* were identified, and the result showed that the *M. dodecandrum* had nine YABBY members, more than the YABBY members in *A. thaliana* (six members), *P. granatum* (six members), *J. curcas* (seven members), and *E. grandis* (six members) [8,18], It may have resulted from the two unique whole genome duplications (WGDs) in *M. dodecandrum* [26]. 

The nine *MdYABBY* genes were unevenly distributed on chromosomes 1, 2, 3, 5, 7, and 9. The analysis of the physicochemical properties of MdYABBYs showed that most YABBY proteins in *M. dodecandrum* were conserved. However, there were two proteins, *MdYABBY5* and *MdYABBY7*, which were significantly different from the others in instability index, showing that these two MdYABBY proteins might play a different role in alternate microenvironments. All MdYABBYs were located in the nucleus, and the result of the subcellular localization analysis was consistent with the website’s prediction, indicating that *MdYABBYs* might play transcriptional regulatory roles in the nucleus, in agreement with previous findings in *Lactuca sativa* [6] and *Glycine max* [14]. 

It is important to infer changes in gene functions and developmental modules to accurately understand the evolutionary history [3,4,28,29]. The results of phylogeny analysis showed that all YABBY genes in *A. thaliana*, *P. granatum*, *J. curcas*, and *E. grandis* could be divided into five clades (FIL, INO, CRC, YABBY2, and YABBY5). Among these clades, clade FIL contained the most of MdYABBYs, as what was reported in rice [11]. In comparison, MdYABBYs were divided into four clades, and no MdYABBY protein was found in clade INO, which might be lost in the interspecific differentiation process. This phenomenon is also found in *L. sativa*, which loses clade YABBY2 [6]. *M. indica* only has three clades, including CRC, YAB5, and YAB3 [22]. In addition, only four clades (CRC/DL, FIL, INO, and YAB2) were found in rice [11]. MdYABBYs expanded in clades FIL, CRC, YABBY2, and YABBY5, which might be related to the two unique WGD events in *M. dodecandrum* [26].

Gene structure is often conserved during evolution [4]. Previous studies have shown that the YABBY gene family contains two typical conserved protein domains, C2C2 zinc finger and YABBY [2,4,5,6]. The amino acid sequence alignment showed that *MdYABBY* genes also contained these two typical conserved protein domains (Figure 2), indicating that the C2C2 zinc finger and YABBY domains were conserved in plant [2,4,5,6]. What’s more, the *MdYABBYs* in the same clade in the phylogenetic tree had the same motifs (Figure 3A), indicating that these genes probably had similar molecular functions. Nevertheless, the expression patterns of *MdYABBYs* showed diversity in different organs in *M. dodecandrum*, such as *MdYABBY 5* and *MdYABBY7*, revealing that *MdYABBYs* may function in different temporal and spatial ways, which is also found in YABBY genes of *Lactuca sativa* (Asteraceae) [6]. This suggests that some YABBY genes in the same subfamily have functional differentiation during evolution.

The cis-acting element is an important part of transcriptional regulation, which is involved in regulating various growth and development mechanisms [4,6]. The cis-acting element analysis showed that *MdYABBYs* contained significant quantities of light-responsiveness elements, indicating that the expression of *MdYABBYs* might be affected by light, consistent with *LsaYABs* [4]. Previous studies found that YABBY genes play an important role in biological processes [2,4,8,16]. In this study, various types of plant regulation cis-acting elements were found in promoter regions of *MdYABBYs*. Abscisic-acid-responsiveness and MeJA-responsiveness elements play an important role in plant abiotic stress and disease resistance [8,24]. What’s more, these two cis-elements are extensively involved in fruit development [4]. In *M. dodecandrum*, abscisic-acid-responsiveness and MeJA-responsiveness elements were present in every *MdYABBYs*, indicating that *MdYABBYs* could respond to various stresses and participate in fruit development. In addition, YABBY genes can regulate the seed development [4,9]. In the present study, the endosperm-expression element was found in *MdYABBY7*, suggesting that it may have similar function. In addition, the number of intron/exon can affect the expression levels of genes, and the genes with less exon number can be induced in a short time than genes with a higher exon number [28]. The intron number of *M. dodecandrum* YABBY genes varied greatly, including two to eight introns, and seven introns were the main structural form of YABBY genes, which was inconsistent with that found in *A. thaliana* [3,4]. *MdYABBY* genes within the same clade had similar motif compositions. Similar results have been identified in *A. thaliana* [3], *A. carambola* [4], *L. sativa* [6], *M. indica* [22], *Juglans regia* and *J. mandshurica* [24], indicating that the YABBY gene family is highly conserved in plant.

In present study, we presented a comprehensive investigation of *MdYABBYs* expression levels in different organs, three flower developmental stages, and three fruit developmental stages based on RT-qPCR and transcriptome data. FAS (clade YABBY2) is critical for fruit size and shape in tomato [30]. Consistent with *FAS*, genes in clade YABBY2 in *M. dodecandrum* were highly expressed during the fruit development. Genes in clade CRC were expressed in reproductive organs, such as carpel and ovule [18]. Here, the expression of the CRC clade was limited to the reproductive organs of *M. dodecandrum*. In *A. thaliana*, FIL-like genes regulate flower development, positively regulate genes involved in plant development and participate in anthocyanin accumulation [21]. *MdYABBY4*, *MdYABBY6*, and *MdYABBY9* were highly expressed in the early stage of flower in *M. dodecandrum*, suggesting that these genes may play a similar role to the FIL gene in *A. thaliana*. Genes in clade YABBY5 were mainly expressed in cotyledons, leaves, and floral organs [18]. In *M. dodecandrum*, the orthologous genes of the YABBY5 clade were highly expressed in the stem, leaf, and early stages of flower development. 

Gene duplication events, such as tandem replication and fragment replication, play an important role in plants evolution processes [4,6,24]. *M. dodecandrum* experienced four WGD events: one γ event shared with most eudicots, one event shared with Myrtales, and two unique events of *M. dodecandrum* [26]. Therefore, we analyzed the duplication patterns of MdYABBY genes. Six pairs of segmental duplicated genes among *MdYABBYs* showed collinear relationships. However, the tandem replication was not found in *MdYABBYs.* Our results suggested that the fragment replication was the major impetus underlying *MdYABBY* genes expansion during evolution, in agreement with the previous finding in *Glycine max* [14]. In the evolution process, genes undergo various selection pressures, and the study of selection pressure can help us to better understand the role of gene evolution [4]. Our results indicated that all *MdYABBYs* underwent strong purifying selection, consistent with the YABBYs of *A. carambola* [4], *J. regia*, and *J. mandshurica* [24], suggesting that their functions might be conserved in evolution processes. The collinearity analysis showed that YABBY genes belonged to the same ancestor. 

## 4. Materials and Methods

### 4.1. Data Sources

The complete genome sequence, genome annotation, protein sequence, coding sequence (CDS), and transcriptome data of *M. dodecandrum* were downloaded from the National Genomics Data Center (NGDC, https://ngdc.cncb.ac.cn, accessed on 16 November 2022) [26]. The genomes of *A. thaliana*, *P. granatum*, and *E. grandis* were downloaded from the National Center for Biotechnology Information (NCBI, https://www.ncbi.nlm.nih.gov/, accessed on 16 November 2022). The protein sequences of YABBY transcription factors in *A. thaliana*, *P. granatum*, *J. curcas*, and *E. grandis* were downloaded from the PlantTFDB plant transcription factor database (http://planttfdb.cbi.pku.edu.cn/, accessed on 16 November 2022).

### 4.2. Identification and Physicochemical Properties Analysis

The YABBY protein sequences of *A. thaliana*, *P. granatum*, *J. curcas*, and *E. grandis* were used as a reference, the local Blast search was performed by TBtools [31], and the candidate genes of YABBY gene family in *M. dodecandrum* were screened at an E-value of <1 × 10^−5^. Then, the Pfam database (http://pfam.Xfam.org/, accessed on 16 November 2022) and NCBI Batch CD-search (https://www.ncbi.nlm.nih.gov/Structure/bwrpsb/bwrpsb.cgi, accessed on 16 November 2022) were used to verify the conserved domain of YABBY proteins [32]. The length of amino acid, the molecular weight, the instability index, the aliphatic index, the grand average of hydropathicity, and the isoelectric point of YABBY proteins in the *M. dodecandrum* were analyzed by ExPasy (http://au.expasy.org/tool.html (accessed on 17 November 2022) [33]. The subcellular localization of the YABBY gene family in *M. dodecandrum* was predicted by CSbio (http://www.csbio.sjtu.edu.cn/bioinf/plant-multi/ (accessed on 18 November 2022)).

### 4.3. Phylogenetic Analysis

To study the phylogenetic relationship of YABBY gene families in *M. dodecandrum*, *A. thaliana*, *P. granatum*, *J. curcas*, and *E. grandis*, multiple sequence alignments of YABBY proteins was carried out by the MUSCLE program of MEGA7 [34]. The maximum-likelihood (ML) method was performed for the phylogenetic analysis. A phylogenetic tree was constructed in the CIPRES Science Gateway web server (RAxML-HPC2 on XSEDE 8.2.10) with 1000 bootstrap replicates [35].

### 4.4. Gene Structure and Conserved Motif Analysis

The gene structures of the YABBY gene family in *M. dodecandrum* were analyzed by using the online tool Gene Structure Display Server (http://gsds.gao-lab.org/, accessed on 16 November 2022) [36]. The conservative motifs of the YABBY gene family in *M. dodecandrum* were searched by online software MEME Suite 4.12.0 (http://meme-suite.Org/ndex.Html, accessed on 16 November 2022) [37]. The maximum number of conserved motifs was set to 10, the length of each motif was set to 6–300 aa, and the E-value was <1 × 10^−20^. The TBtools was used for the visualization of the results [31].

### 4.5. Cis-Acting Element Prediction

The DNA sequence of the 2000 bp upstream of the YABBY gene initiation codon (ATG) was extracted from the *M. dodecandrum* genome by TBtools [31]. Then, the sequences were analyzed by PlantCARE (http://bioinformatics.psb.ugent.be/webtools/plantcare/html/ (accessed on 18 November 2022)) [38], and the result was visualized by TBtools [31].

### 4.6. Gene Location and Collinearity Analysis

The location of the YABBY gene family of *M. dodecandrum* was extracted from gff3 files in genome data, and then, the distribution map of the YABBY gene family was drawn by TBtools [31]. A simple Ka/Ks calculator was used in TBtools. To analyze the collinear relationships among *M. dodecandrum*, *A. thaliana*, *P. granatum*, and *E. grandis*, genome sequence files and GFF files of these three species were used. The one-step MCScanX function of TBtools was used to analyze the collinear relationship, and the Advenced Circos and Multiple Synteny Plot of TBtools were used for visualization [31].

### 4.7. RNA Extraction and cDNA Synthesis

A plant material was collected from wild *M. dodecandrum*, which was located in the mountains of the Soil and Water Conservation Garden of Fujian Agriculture and Forestry University (N26°51′33″, E119°14′42″). The total RNA of the tender leaf, tender root, tender stem, small fruit, medium fruit, big fruit, flower bud, medium flower, and mature flower of *M. dodecandrum* was extracted by a FastPure ^®^ Plant Total RNA Isolation Kit (Vazyme, Nanjing, China). The concentration and OD_260_/OD_280_ were determined by a NanoDrop 2000 spectrophotometer (Thermo Scientific, Waltham, MA, USA), and the integrity of RNA was detected by agarose gel electrophoresis. A HiScript III 1st Strand cDNA Synthesis Kit (+gDNA wiper; Vazyme, Nanjing, China) was used to reverse transcribe RNA into cDNA.

### 4.8. Expression Pattern and Network Analysis

The expression patterns of YABBY genes were analyzed by using RNA-seq data from different tissues of *M. dodecandrum*. After standardizing the data, TBtools was used to draw the heatmap of *MdYABBYs* [31]. To further analyze the expression patterns of the YABBY gene family in *M. dodecandrum*, RT-qPCR was performed. The RT-qPCR primers were designed by Primer3Plus (https://www.primer3plus.com/, accessed on 17 November 2022); Supplementary Table S1). A Taq Pro Universal SYBR qPCR Master Mix kit (Vazyme, Nanjing, China) was used for the RT-qPCR analysis. The *MdActin* gene was used as an internal reference [32], and the biological repetition and technical repetition were performed three times. The expression level was calculated by the 2^−∆Ct^ method [1,32]. 

### 4.9. Cloning and Subcellular Localization Analysis of MdYABBYs

Firstly, specific primers were designed according to the CDS sequence of *MdYABBYs* by Snapgene 3.2.1, removing the stop codon and adding the *XbaI* and *KpnI* restriction sites. Then, the first strand cDNA of *M. dodecandrum* leaves was used as a DNA template for PCR amplification, and the PCR products were separated by 1.2% agarose gel electrophoresis and purified with a FastPure Gel DNA Extraction Mini Kit (Vazyme, Nanjing, China). The purified product was ligated to the pMDC202 vector and transformed into Escherichia coli (DH5α) by a ClonExpress^®^ Ultra One Step Cloning Kit (Vazyme, Nanjing, China). The positive clones were selected and sequenced by Sangon Biotech (Shanghai, China) Co., Ltd. The correct clone was extracted from the plasmids by FastPure Plasmid Mini Kit (Vazyme, Nanjing, China). After that, the plasmid of *MdYABBYs* was transferred to agrobacterium tumefaciens (GV3101) by the freeze-thaw method. Finally, the 35S::MdYABBYs−GFP vector and the vector without the genes were transferred into tobacco leaf separately. After 48 h, GFP fluorescence signals were observed by a confocal laser scanning microscope (LSM710; CarlZeiss, Jena, Germany). The primers of *MdYABBYs* are listed in Supplementary Table S1.

## Figures and Tables

**Table 1:** Physicochemical properties analysis and subcellular localization of the YABBY gene family in *M. dodecandrum*.

Gene ID	Gene Name	Isoelectric Point (PI)	Molecular Weight (Average)	Number of Amino Acids	Instability Index	Aliphatic Index	Grand Average of Hydropathicity (GRAVY)	Subcellular Localization
DR001430	*MdYABBY1*	8.69	19,545.35	175	45.72	74.17	−0.270	Nucleus
DR012482	*MdYABBY2*	9.66	29,200.37	263	57.98	79.77	−0.455	Nucleus
DR031028	*MdYABBY3*	9.04	20,517.28	187	55.66	74.12	−0.457	Nucleus
DR030078	*MdYABBY4*	8.42	24,425.01	220	49.65	74.91	−0.358	Nucleus
DR033281	*MdYABBY5*	8.50	14,360.52	128	38.97	60.94	−0.521	Nucleus
DR031379	*MdYABBY6*	8.71	24,915.46	222	55.56	68.96	−0.563	Nucleus
DR021453	*MdYABBY7*	8.95	21,037.75	196	39.80	60.20	−0.517	Nucleus
DR024191	*MdYABBY8*	9.37	20,564.48	188	50.57	76.86	−0.386	Nucleus
DR023493	*MdYABBY9*	8.70	23,946.49	215	53.26	73.95	−0.387	Nucleus

**Table 2:** Selective pressure analysis of *MdYABBYs*.

Sequence 1	Sequence 2	Ka	Ks	Ka/Ks
*MdYABBY1*	*MdYABBY2*	0.15832754	0.42003242	0.37694125
*MdYABBY3*	*MdYABBY8*	0.03311535	0.30167713	0.10977084
*MdYABBY4*	*MdYABBY6*	0.04780839	0.53613362	0.08917253
*MdYABBY4*	*MdYABBY9*	0.02688089	0.13608643	0.19752809
*MdYABBY5*	*MdYABBY7*	0.15915999	0.91519305	0.17390865
*MdYABBY6*	*MdYABBY9*	0.03946229	0.49981556	0.07895371

## Data Availability

Not applicable.

## Supplementary Materials

The following are available online at https://www.mdpi.com/article/10.3390/ijms24044174/s1.
